# PTEN Gene: A Model for Genetic Diseases in Dermatology

**DOI:** 10.1100/2012/252457

**Published:** 2012-04-30

**Authors:** Corrado Romano, Carmelo Schepis

**Affiliations:** ^1^Unit of Pediatrics and Medical Genetics, I.R.C.C.S. Associazione Oasi Maria Santissima, 94018 Troina, Italy; ^2^Unit of Dermatology, I.R.C.C.S. Associazione Oasi Maria Santissima, 94018 Troina, Italy

## Abstract

PTEN gene is considered one of the most mutated tumor suppressor genes in human cancer, and it's likely to become the first one in the near future. Since 1997, its involvement in tumor suppression has smoothly increased, up to the current importance. Germline mutations of PTEN cause the PTEN hamartoma tumor syndrome (PHTS), which include the past-called Cowden, Bannayan-Riley-Ruvalcaba, Proteus, Proteus-like, and Lhermitte-Duclos syndromes. Somatic mutations of PTEN have been observed in glioblastoma, prostate cancer, and brest cancer cell lines, quoting only the first tissues where the involvement has been proven. The negative regulation of cell interactions with the extracellular matrix could be the way PTEN phosphatase acts as a tumor suppressor. PTEN gene plays an essential role in human development. A recent model sees PTEN function as a stepwise gradation, which can be impaired not only by heterozygous mutations and homozygous losses, but also by other molecular mechanisms, such as transcriptional regression, epigenetic silencing, regulation by microRNAs, posttranslational modification, and aberrant localization. The involvement of PTEN function in melanoma and multistage skin carcinogenesis, with its implication in cancer treatment, and the role of front office in diagnosing PHTS are the main reasons why the dermatologist should know about PTEN.

## 1. PTEN Gene: What It Is and How It Works

PTEN stands for phosphatase and tensin homolog deleted in chromosome 10, and it is considered one of the most mutated tumor suppressor genes in human cancer. In the near future, it is likely to become the first one overcoming the current leader, p53 gene [1]. The involvement of PTEN's alteration in tumorigenesis has been first suspected and subsequently proven in 1997 [2], when high frequency of loss of heterozygosity (LOH) at 10q23 chromosome band was observed in several human tumors. Furthermore, the suppression of tumorigenesis in glioblastoma murine cells by the wildtype chromosome 10 led to envision a tumor suppressor gene mapping in 10q23. Such gene was eventually isolated by the above-mentioned authors and called PTEN. They detected homozygous deletions, frame shift, or nonsense mutations in PTEN in 63% (5/8) of glioblastoma cell lines, 100% (4/4) of prostate cancer cell lines, and 10% (2/20) of breast cancer cell lines. Steck et al. [3] independently isolated the same gene and called it mutated in multiple advanced cancers-1 (MMAC-1). Indeed, a common feature of PTEN somatic mutations, already presented in 10q LOH, is the association with advanced-stage tumors (mainly glial and prostate cancers), whereas this is not true for endometrial cancer, being affected equally at all the stages. This has led to the suggestion that the activation of PTEN is at an early stage in endometrial carcinogenesis, but later on in glial and prostatic carcinogenesis. This mechanism is the cornerstone of the classical two-hit Knudson' hypothesis [4]: a single mutation in one homolog of a tumor-suppressor gene is not sufficient to initiate tumor growth; however, deletion or disabling of the allele on the homologous chromosome results in unregulated cell growth. Both sporadic and hereditary tumors can be explained by such mechanism. In sporadic tumors, both alleles are normal at conception; subsequently, a postzygotic mutation (first hit) in one cell creates the heterozygosity (one mutant and one normal allele); thereafter, a deletion or a new mutation (second hit) in the other allele of that cell provokes the LOH, starting the uncontrolled tumor growth. In hereditary tumors, the heterozygosity for mutant allele (first hit) is present at conception, and is sufficient that a postzygotic mutation (second hit) during life creates the LOH for the onset of uncontrolled tumor growth.

Liaw et al. [5] found germline mutations of PTEN gene in families with Cowden syndrome [6] (CS), showing the function of tumor suppressor gene also in the germline. Furthermore, germline PTEN mutations lead to increased breast cancer incidence, but do not frequently cause familial breast cancer [7], notwithstanding 10% of breast cancer cell lines have inactivated PTEN [2, 3]. Recently it has been shown that PTEN loss is a common event in breast cancers caused by BRCA1 deficiency [8]. Marsh et al. [9] defined PTEN hamartoma tumor syndrome (PHTS) as a syndromic condition including one or more hamartomas which has its biological basis in a germline mutation of the PTEN gene. Following such assumption, PHTS includes patients with the previous diagnosis of CS, Bannayan-Riley-Ruvalcaba syndrome [10] (BRRS), Proteus syndrome [11] (PS), Proteus-like syndrome [12] (PLS), and Lhermitte-Duclos syndrome [13] (LDS).

 Li et al. [2] have shown that PTEN gene is a human cdc14 homolog, like CDC14A and CDC14B. The cdc14 gene is a key point for the progression of cell cycle in *Saccharomyces cerevisiae*. Its protein acts in late nuclear division preparing for subsequent DNA replication. The human PTEN gene spans 103,207 bases, is made up of 9 exons, and codes for a 1212-bp transcript and a 403-amino-acid protein.

 The PTEN product has the kinetic properties of dual-specific phosphatases [14] and acts on G1 cell cycle progression through negative regulation of the PI3-kinase/Akt or PKB signalling pathway [15]. PTEN is a member of the protein-tyrosine phosphatase (PTP) gene superfamily [2, 3]. These are genes consisting of conserved catalytic domains, flanked by noncatalytic regulatory sequences [16]. The PTP catalytic domains show a “signature motif,” which is the canonical sequence HCXXGXXRS/T. Among the PTP superfamily genes, a further split is made in “classic” PTP (acting only towards phosphothyrosine residues) and dual-specificity phosphatase families (dephosphorylating phosphotyrosine, phosphoserine and/or phosphotreonine). PTEN is a dual-specificity phosphatase. The catalytic domain of PTEN has been proven to be essential for its function, which is lost following any mutation within the signature motif [17].

 PTEN gene in glioblastoma-derived cell lines regulates hypoxia- and IGF-1-induced angiogenic gene expression by regulating Akt activation of HIF-1 activity [18]. Restoration of wild-type PTEN to glioblastoma cell lines lacking functional PTEN ablates hypoxia and IGF-1 induction of HIF-1-regulated genes. In addition, Akt activation leads to HIF-1*α* stabilization, whereas PTEN attenuates hypoxia-mediated HIF-1 *α* stabilization. Loss of PTEN during malignant progression contributes to tumor expansion through the deregulation of Akt activity and HIF-1-regulated gene expression. PTEN abnormalities have been found also in primary acute leukemias and non-Hodgkin's lymphomas [19].

 PTEN and phosphorylated Akt levels are inversely correlated in the large majority of the examined samples, suggesting that PTEN regulates phosphatidylinositol 3,4,5-triphosphates and may play a role in apoptosis. Overexpression of PTEN inhibits cell migration, whereas antisense PTEN enhances migration [20]. The phosphatase domain of PTEN is essential because its inactivation does not allow the downregulation of integrin-mediated cell spreading and formation of focal adhesions, peculiar of wild-type PTEN. Overexpression of focal adhesion kinase (FAK) partially antagonizes the effects of PTEN. Thus, the negative regulation of cell interactions with the extracellular matrix could be the way PTEN phosphatase acts as a tumor suppressor.

 PTEN gene plays an essential role in human development. Indeed, the additional effect of three homozygotic mutations together (e.g., mutations in both alleles) produces early embryonic lethality in mice, whereas heterozygosis (e.g., mutations in one allele) increases tumor incidence [21–23]. Furthermore, PTEN antagonizes growth factor-induced Shc phosphorylation and inhibits the MAP kinase (MAPK) signalling pathway [24]. The way this inhibition is accomplished is currently understood as a suppression by the PTEN protein phosphatase activity [25].

**Figure 1:**
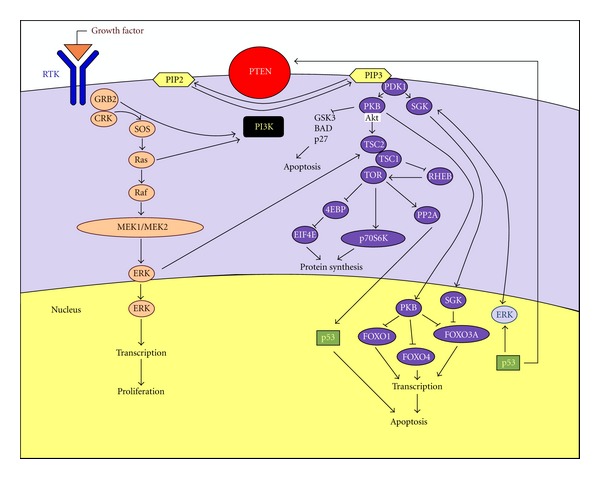
Pathway involving the PTEN protein.

The function of PTEN gene and the way its mutation can cause a disease can barely be understood if one does not see the PTEN protein inside the PI3K/Akt/mTOR signalling pathway [26] (Figure 1). The activation of phosphoinositide 3′ kinase (PI3K) is the primary event in this pathway [27]. This can occur from several growth factor receptors (GFRs), such as PDGFR, EGFR, FGFR, IGF-1R, VEGFR, IL-R, interferon receptors (IF-Rs), integrin receptors, and the Ras pathway [28–30]. The major role of PI3K is the phosphorylation of phosphatidylinositol (4,5) P (PIP2) to phosphatidylinositol (3,4,5) P (PIP3). PIP3 binds and translocates Akt near the cell membrane where it can be phosphorylated and activated by phosphatidylinositol(3,4,5)P-dependent kinase 1 (PDK1) and phosphatidylinositol(3,4,5)P-dependent kinase 2 (PDK2). Akt has several downstream effectors which mediate its ability to promote cell survival and growth. Then, activation of PI3K/Akt pathway is observed in several human cancers. PTEN is the antagonist of PI3K because it dephosphorylates PIP3 to PIP2. The PI3K/PTEN imbalance, caused for instance by a mutation of PTEN, is then responsible for the progression to human cancer.

Salmena et al. [1] have recently proposed a model for the causes and consequences of PTEN loss. While retinoblastoma (RB) gene has been the foundation of the Knudson's hypothesis [4], showing that only the homozygous loss of such gene can start the retinoblastoma initiation, PTEN behaves in a different way. There is compelling evidence in mice confirming PTEN as a haploinsufficient tumor suppressor gene: loss of one allele leads to the progression of a lethal polyclonal autoimmune disorder [31]; epithelial cancers, such as prostate cancer, are driven by PTEN heterozygosity [32]; cellular levels of PTEN protein inversely correlate with the occurrence of invasive prostate cancer [1]. Consequently, functional loss of one PTEN allele is critical for the onset of cancer in mice. Things in humans are little less compelling, but the evidence is growing. The association between PTEN heterozygous germline mutations and the so-called PHTS is the first proof. Further evidence for haploinsufficiency is supported by the following observations: some tumors arisen in patients with CS do not show biallelic mutations of PTEN gene [33]; primary prostate cancers are associated with loss or alteration of one PTEN allele in 70% of cases [34], whereas homozygous deletion is present in 10% of cases [35]; the occurrence of monoallelic mutation of PTEN in breast cancer is much more frequent (30–40% versus 5%) than that of biallelic loss [36–38]. Besides the above-mentioned proofs of the contribution of PTEN haploinsufficiency to tumor progression and cancer syndromes, the identification of CS and tumor-derived PTEN mutations preserving partial or full PTEN lipid phosphatase function [39] allows the notion that even minor impairments of PTEN function can lead to cancer. The model of Santena et al. [1] springs from the above results: the loss of PTEN function, caused not only by classical genetic mutations leading to heterozygous (50% of function) or homozygous (0% of function) loss, but also by other molecular mechanisms, such as transcriptional regression, epigenetic silencing, regulation by microRNAs, posttranslational modification, and aberrant localization, can lead to subtle and/or dramatic losses of PTEN function, behaving as a stepwise gradation of function. An unexpected result has been put forward by Chen et al. [40], who studied the relationship between PTEN dose and tumor progression in mouse models of prostate specific loss of PTEN: complete acute loss of PTEN promoted a strong senescence response opposing tumor progression. Campisi and d'Adda di Fagagna [41] interpreted senescence as an antitumor mechanism set off by tumor suppressor genes in response to triggers including DNA damage and oncogene activation.

While heterozygous and homozygous mutations are now widely understood, the other molecular mechanisms deserve some clarification. We will do so, showing that all these mechanisms have a significant impact in the way PTEN functions, as a model of tumor suppressor gene.

While transforming growth factor *β* (TGF*β*) was considered the single transcriptional regulator of PTEN gene in 1997 [42], acting in a negative way, today the story is much more complicated. Several factors have been discovered to upregulate, such as early growth-regulated transcription factor-1 (EGR-1) [43] which is a downstream effector of Insulin-like growth factor 2 (IGF-2) [44], and peroxisome proliferation-activated receptor-*γ* (PPAR-*γ*) [45], p53 [46], and MYC [47], or downregulate PTEN, such as c-Jun [48], NF*κ*B [49], and HES-1 [47]. Furthermore, a peculiar way of PTEN's transcriptional regulation is that of NOTCH1, which increases the transcription of PTEN at least in two fashions: activating MYC [47] or repressing CBF-1 [50, 51], which is a downregulator of PTEN. Conversely, NOTCH1 represses PTEN's transcription, activating the known PTEN's downregulator HES-1 [47]. Another complication of the action of NOTCH1 on PTEN is the tissue specificity, which allows upregulation or downregulation according to the involved tissues.

Epigenetic silencing means that the function of a gene is broken, not by means of DNA mutations, but for other mechanisms, which are mainly the DNA methylation of the gene promoter and the histone modification. The promoter methylation of PTEN gene has been associated to several cancers [52–54].

MicroRNAs (miRNAs) are single-stranded RNAs, made up of a few (usually 22) nucleotides, which repress the mRNA translation. One of the most studied miRNAs, miR-21, has been reported as a repressor of PTEN, exerting its oncogenic activity, at least partially, downregulating PTEN expression [55–59].

Posttranslational modification is a further way of regulation of the action of a gene, without any change in its DNA. It can be defined as the chemical modification of a protein after its translation. The four main chemical reactions are phosphorylation, acetylation, oxidation, and ubiquitination. PTEN has six phosphorylation sites [1], which have been involved in the modulation of its tumor suppressor functions, subcellular distribution, and stability. They are Threonine 366 (Thr366), Serine 370 (Ser370), Ser385, Ser380, Thr382, and Thr383. The last three are collectively referred to as the STT cluster, which has the most important effects on PTEN function. The wild-type or phosphorylated STT cluster is maintained to stabilize PTEN in a closed state, whereas the mutation at these phosphorylation sites opens the protein conformation, making it less stable [60]. A dephosphorylation of the STT cluster is currently considered a common way leading to cancer activation [61]. PTEN interacts with the nuclear histone acetyltransferase-associated PCAF protein, promoting PTEN acetylation at Lysine 125 (Lys125) and Lys128 sites, which in turn negatively regulates PTEN catalytic activity [62]. What is now called reactive oxygen species (ROS), which is made of oxygen ions, free radical, and peroxides, are very small molecules sharing a high reactivity due to presence of unpaired valence shell electrons. ROS modulates PTEN catalytic activity by oxidative-stress-induced formation of a disulfide bond between the active site cysteine 124 (Cys124) and Cys71 [63]. Ubiquitin [64] is a highly conserved regulatory protein that is widely (ubiquitously) expressed in eukaryotes. Ubiquitination refers to the posttranslational modification of a protein by the covalent attachment (via an isopeptide bond) of one or more ubiquitin monomers. The most prominent function of ubiquitin is labeling proteins for proteasomal degradation. Proteasome inhibition increases the half-life of PTEN [65], and the exposure of human bronchial cells to zinc ions promotes ubiquitin-dependent degradation of PTEN [66], but inhibitors of the proteasome may destabilize PTEN [67]. Notwithstanding such incoherent results, ubiquitination to lysine 13 (Lys13) and Lys289 is needed for the nuclear-cytoplasmic shuttling of PTEN [68]. Such last fact implies the notion that PTEN protein can move from cytoplasm to nucleus, and backward. However, this was not clear at the very beginning, when everyone thought it was exclusively localized to the cytoplasm. PTEN is abundantly localized in the nucleus of primary, differentiated, and resting cells, while there is a sharp decrease in cancer cells [69–72]. Cell cycle stage and differentiation status are, consequently, related to PTEN localization. The fact that PTEN protein is localized in the nucleus, besides the cytoplasm, has an important role in the tumor suppressor function of such protein. This is proven by the report of patients with more aggressive tumors, such as esophageal squamous cell carcinoma [73], cutaneous melanoma [74, 75], colorectal cancer [76], and pancreatic islet cell tumors [72], having their PTEN protein absent in the nucleus.

## 2. Why Should the Dermatologist Know about PTEN?

 Besides using PTEN as a model for genetic disease, the dermatologist should deepen his knowledge of such fascinating pathway for some very practical reasons.

First of all, think of melanoma. Approximately 70% of melanomas have elevated Akt3 signaling both for increased gene copy number and PTEN loss. Consequently, the targeting of (V600E)B-Raf and Akt3 signalling can prevent or treat cutaneous melanocytic lesions. The development of agents specifically targeting these proteins would be very useful, because they have fewer side effects than those inhibiting both normal and mutant B-Raf protein or targeting all three Akt isoforms. Recently [77], a nanoliposomal-ultrasound-mediated approach reported for delivering small interfering RNA (siRNA) specifically targeting (V600E)B-Raf and Akt3 into melanocytic tumors present in skin to retard melanoma development. Novel cationic nanoliposomes stably encapsulate siRNA targeting (V600E)B-Raf or Akt3, providing protection from degradation and facilitating entry into melanoma cells to decrease expression of these proteins. Low-frequency ultrasound using a lightweight four-cymbal transducer array enables penetration of nanoliposomal-siRNA complex throughout the epidermal and dermal layers of laboratory-generated or animal skin. Nanoliposomal-mediated siRNA targeting of (V600E)B-Raf and Akt3 led to a cooperatively acting approximately 65% decrease in early or invasive cutaneous melanoma compared with inhibition of each singly with negligible associated systemic toxicity. Thus, cationic nanoliposomes loaded with siRNA targeting (V600E)B-Raf and Akt3 provide an effective approach for targeted inhibition of early or invasive cutaneous melanomas. Furthermore, it is currently thought that the progression of human cutaneous melanomas behaves in a stepwise fashion, due to accumulating genetic and epigenetic alterations. The combination of PTEN deficiency and Braf activation induces a melanoma *in-situ*-like phenotype without dermal invasion. Further addition of cell autonomous TGF-*β* activation in the context of PTEN deficiency and Braf activation promotes dermal invasion in skin cultures without significantly promoting proliferation *in vitro* and *in vivo*. This proinvasive phenotype of cell autonomous TGF-*β* activation is genetic context dependent, as hyperactivating the TGF-*β* type I receptor without PTEN deficiency and Braf activation failed to induce an invasive behavior. Evidence of genetic interactions among PTEN deficiency, Braf activation, and cell autonomous TGF-*β* activation shows that distinct stages of human melanoma are genetically tractable in the proper tissue architecture [78].

Secondly, multistage skin carcinogenesis is prone to gene synergism. The ablation of PTEN function in mouse epidermis expressing activated Fos leads to hyperplasia, hyperkeratosis, and tumors that move forward to highly differentiated keratoacanthomas, rather than to carcinomas [79].

Thirdly, the dermatologist can be the front office in diagnosing a PHTS. Acral papular neuromatosis [80] and mucocutaneous neuromas [81] can be the first sign of a disease caused by a PTEN mutation. Katona et al. [82] analysed a series of patients affected by mycosis fungoides, showing that LOH studies are a robust method for evaluating genetic abnormalities in mycosis fungoides, and several loci associated with the PTEN appear to be associated with progression from plaque to tumor stage. It has been shown that PTEN was significantly higher in depigmented epidermis, implying that vitiliginous keratinocytes may be more susceptible to TNF-alpha-mediated apoptosis through impaired Akt and NF-kappaB activation. Keratinocytes showing impaired Akt activation demonstrated increased apoptosis with less activation of NF-kappaB. Thus, reduced activation of NF-kappaB via impaired PI3K/Akt activation under increased TNF-alpha levels could result in increased apoptosis of vitiliginous keratinocytes [83].

Finally, the pathogenic role of mutated PTEN involves some conditions collectively called PTEN hamartoma tumor syndrome (PHTS) [84, 85], such as Cowden syndrome [86], Bannayan-Riley-Ruvalcaba syndrome (BRRS) [87], and Proteus syndrome (PS) [88].

**Figure 2:**
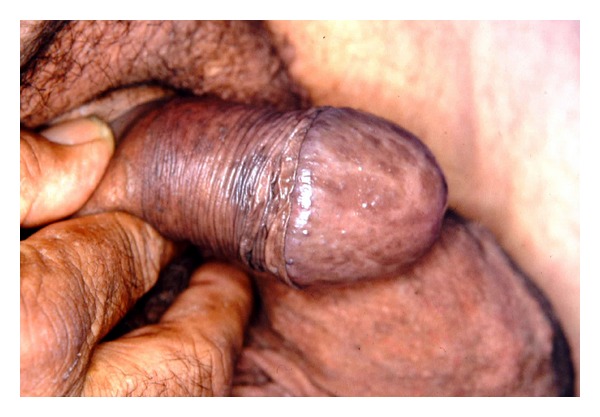
Speckled penis in a patient affected by Bannayan-Riley-Ruvalcaba syndrome.

**Figure 3:**
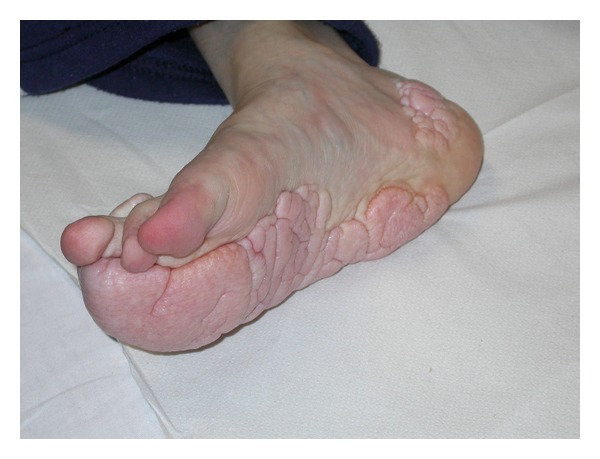
Cerebriform plantar hyperplasia in a girl affected by Proteus syndrome.

Such rare diseases highlight tumoral degeneration in many body organs. Affected patients show usually variable degrees of intellective disability. The cutaneous surface of PHTS patients leads one to suspect the disease being present, many times in a peculiar way. Main examples are speckled penis in BRRS [87] (Figure 2), plantar cerebriform hyperplasia in 70–80% of PS patients [88, 89] (Figure 3), and three or more trichilemmomas in CS [86].

The spectrum of clinical findings associated with PTEN tumor suppressor gene germline mutations includes also mucocutaneous neuromas, as reported by Shaffer et al., who stated that this is an underrecognized manifestation of the gene [81].

These are only some reasons why the knowledge of PTEN is very much needed for the dermatologist, not only for his/her culture, but also for practical issues, such as a targeted therapy in the near future.
